# Early glucose metabolism in children at risk for type 1 diabetes based on islet autoantibodies compared to low-risk control groups

**DOI:** 10.3389/fendo.2022.972714

**Published:** 2022-09-12

**Authors:** Olli Helminen, Tytti Pokka, Susanna Aspholm, Jorma Ilonen, Olli Simell, Mikael Knip, Riitta Veijola

**Affiliations:** ^1^ Department of Pediatrics, PEDEGO Research Group, Medical Research Center, Oulu University, Hospital and University of Oulu, Oulu, Finland; ^2^ Surgery Research Unit, Cancer and Translational Medicine Research Unit, Medical Research Center Oulu, University of Oulu and Oulu University Hospital, Oulu, Finland; ^3^ Tampere Centre for Child Health Research, Tampere University Hospital, Tampere, Finland; ^4^ Immunogenetics Laboratory, University of Turku, Turku, Finland; ^5^ Department of Pediatrics, University of Turku and Turku University Hospital, Turku, Finland; ^6^ Pediatric Research Center, New Children’s Hospital, Helsinki University Hospital, Helsinki, Finland; ^7^ Faculty of Medicine, University of Helsinki, Helsinki, Finland

**Keywords:** Type 1 diabetes, glucose metabolism, Islet autoantibodies, preclinical, HbA1c, random plasma glucose, oral glucose tolerance test (OGTT)

## Abstract

**Background:**

Anatomic variation or early differences in glucose metabolism have been linked to the development of type 1 diabetes. We aimed to describe early glucose metabolism based on HbA1c, oral glucose tolerance test (OGTT), and random plasma glucose years before the presentation of type 1 diabetes in five risk groups based on autoantibody combinations. For the first time, we were able to include for comparison children with very low risk of progression to type 1 diabetes.

**Methods:**

The Finnish Diabetes Prediction and Prevention birth cohort study screened newborn infants for HLA susceptibility to type 1 diabetes since 1994. Those carrying a risk genotype were prospectively followed up with islet autoantibody testing. Glucose parameters were obtained starting from the time of seroconversion. By 31 August 2014, 1162 children had developed at least one islet autoantibody and were included in the current study. Type 1 diabetes was diagnosed in 335 children (progressors). In the non-progressor groups, 207 developed multiple (≥2) biochemical islet autoantibodies, 229 a single biochemical autoantibody, 370 ICA only, and 64 transient autoantibodies. Children were divided into five risk groups. Glucose metabolism was evaluated.

**Results:**

We observed lower HbA1c values in early follow-up 4.5 to 6.0 years before diagnosis in the progressors when compared to the same time in children with a single biochemical autoantibody or low-risk (ICA only and transient) participants, who did not progress to clinical type 1 diabetes. However, no such differences were observed in OGTTs or random plasma glucose. The variation was minimal in glucose values in the low-risk groups.

**Conclusion:**

We report the possibility of early alteration in glucose metabolism in future progressors. This could suggest early defects in multiple glucose-regulating hormones.

## Introduction

Type 1 diabetes is a common chronic disease usually appearing during childhood. The incidence has been rising throughout the Western world (1), being highest in Finland (2). The presentation of type 1 diabetes is in most cases acute with the presence of typical symptoms, such as polyuria, polydipsia, and fatigue. Approximately 20%–30% of patients have diabetic ketoacidosis at diagnosis, but if the child is participating in regular follow-up before diagnosis, ketoacidosis is relatively rare (3). The Human Leukocyte Antigen (HLA) region accounts for a major part of the genetic risk for type 1 diabetes (4), but a number of non-HLA genes are also involved (5). If multiple (≥2) islet autoantibodies are present in the peripheral circulation, the individual is likely to develop type 1 diabetes, with a 70% risk during follow-up for 10 years (6). Glucose abnormalities occurred approximately 2 years before diagnosis when comparing children positive for multiple autoantibodies who developed overt disease and those who did not (7). The commonly used glucose parameters that can improve the prediction of the time to diagnosis include HbA1c (8), oral glucose tolerance test (OGTT) (9), random plasma glucose (9), C-peptide (10), and intravenous glucose tolerance test (IVGTT) (11).

A series of intervention studies aimed at preservation of the endogenous insulin secretion have been performed in patients with recent-onset type 1 diabetes over the years with no longstanding results (12). Multiple explanations for this lack of success have been implicated, such as heterogeneity of the disease process (13) and different triggering events (14). Recently a possible role of a defect or disorder in early organogenesis of the pancreas has been proposed, since in patients with newly diagnosed type 1 diabetes the mass of the pancreas is significantly reduced when compared to healthy controls, possibly predisposing to undesirable immune reactions (15, 16). Also in IVGTT studies baseline first-phase insulin response (FPIR) values seem to be decreased in children progressing to type 1 diabetes as early as 4–6 years before diagnosis suggesting early β-cell dysfunction (11, 17). Previous studies on glucose metabolism have focused on high-risk children based on autoantibodies, but large-scale cohorts of glucose values in low-risk children are lacking.

In the current study we wanted to analyze the differences in glucose metabolism in five different risk groups defined by clinical disease and islet autoantibody combinations. For the first time we were able to include two very low-risk groups of children: those with islet cell antibodies (ICA) only and those with transient autoantibodies, and compare the baseline values of HbA1c, OGTT, and random plasma glucose. We hypothesized that an early defect in glucose parameters could be present already years before disease onset.

## Materials and methods

### Study design

**Figure 1:**
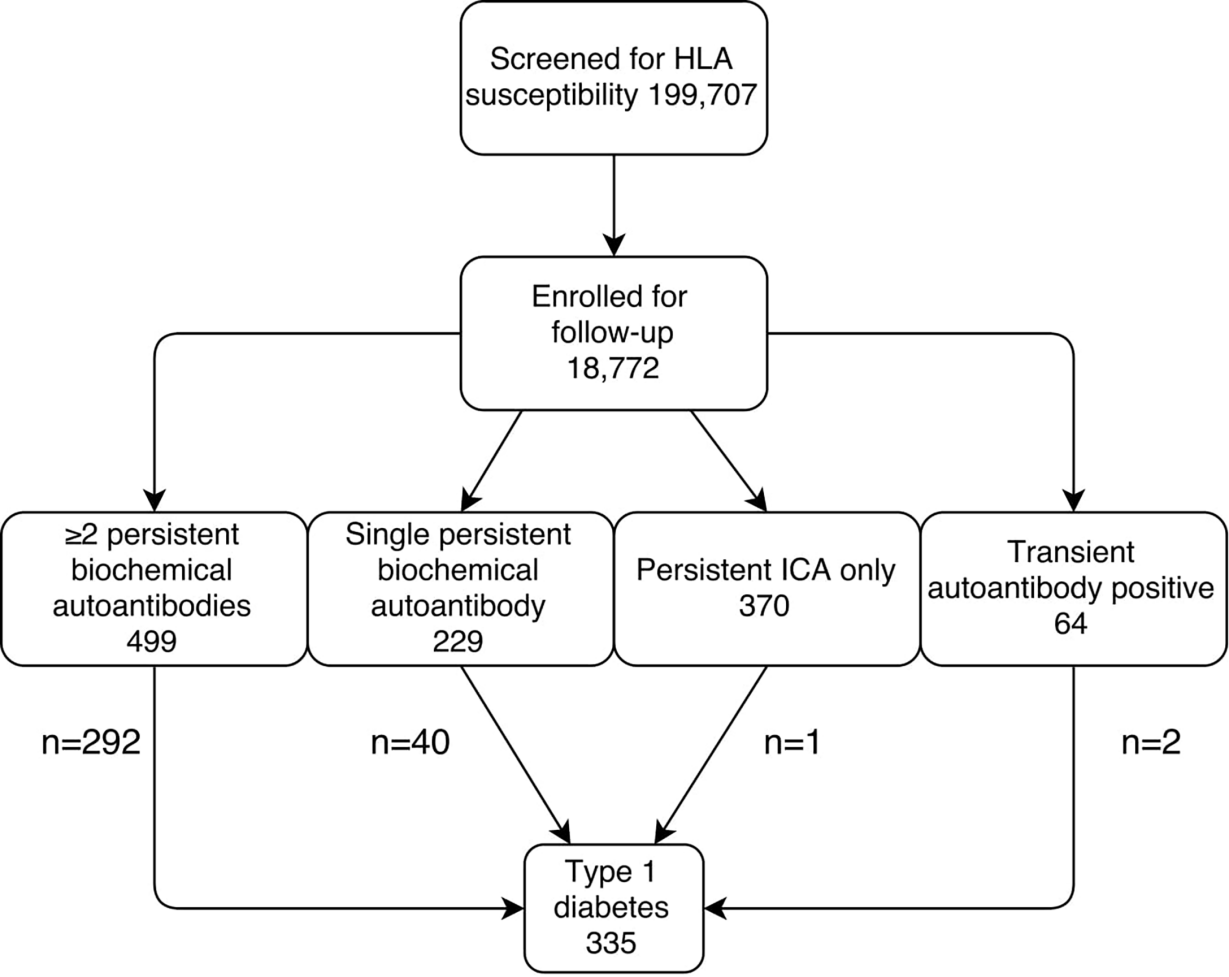
Flow-chart for the identification of the current study groups. The follow-up ended on August 31, 2014.

The Diabetes Prediction and Prevention (DIPP) project is a Finnish birth cohort study in which newborn infants from the University Hospitals in Oulu, Tampere, and Turku are screened for HLA susceptibility to type 1 diabetes after parental consent. The recruitment started in 1994 and is still going on. Families with a child carrying a risk HLA genotype were invited to prospective follow-up at 3–12-month intervals until the age of 15 years or until the development of clinical disease. Islet autoantibodies were analyzed at every visit, including ICA and autoantibodies to insulin (IAA), glutamic acid decarboxylase (GADA), and islet antigen 2 (IA-2A). If seroconversion for any of these occurred subsequent visits were scheduled every 3 months, and further if seroconversion to ≥2 autoantibodies (including ICA) was detected, monitoring of HbA1c, OGTT, and random plasma glucose started. HbA1c and random plasma glucose were analyzed at every 3-month visit and OGTT was performed once a year. Seroconversion to positivity was confirmed in a subsequent sample. In the Oulu University Hospital HbA1c and random plasma glucose were analyzed even when a single autoantibody was detected, providing a low-risk control group characterized by ICA only. Type 1 diabetes diagnosis was based on typical symptoms and high plasma glucose, or two separate diabetic OGTT tests in asymptomatic patients as WHO recommends (18). The inclusion criteria in the current study were DIPP participants with informed parental consent and at least one glucose parameter measured during the follow-up. This has been mentioned in **Figure 1**. There were no exclusion criteria. The follow-up for this study ended by August 31, 2014. The DIPP study has been approved by the Ethics Committees in the participating universities and hospital districts (Turku University Ethics Committee, 10/1994 §228). All families participating in the study have provided written informed consent.

### Immunological screening

Seroconversion was defined as the time when the first autoantibody appeared for the first time and multiple (≥2) autoantibodies when two or more were detected for the first time (including ICA). Positivity for multiple persistent biochemical autoantibodies was defined as positivity for two of IAA, GADA, and IA-2A in at least two subsequent samples. Single persistent biochemical autoantibody was defined as positivity for one of IAA, GADA, or IA-2A in at least two susbsequent samples with no other biochemical autoantibodies appearing during follow-up. Low-risk groups of ICA only and transient autoantibody positivity were defined as persistent positivity to ICA without any other autoantibodies, and as a single positive measurement (ICA, IAA, GADA, or IA-2A), followed by negative samples. Autoantibody analyses were performed as described previously (19). The cut-off limits for autoantibody positivity have been reported previously (9). In the Diabetes Autoantibody Standardization (DASP) workshop in 2005 the following sensitivities and specificities have been reported: IAA 58% and 96%, GADA 82% and 96%, and IA-2A 72% and 100%, respectively.

### Genetic screening

HLA-conferred susceptibility was analyzed using cord blood as described earlier (20). According to various HLA DRB1–DQA1–DQB1 haplotype combinations six risk groups were identified: high, moderately increased, slightly increased, neutral, slightly decreased, and strongly decreased (20).

For the current study, risk groups of neutral, slightly decreased risk, and strongly decreased risk were combined due to the low number of children in these groups, and the analyses were performed with four risk groups.

### HbA1c assays

In Oulu University Hospital an immunoassay-based method was applied throughout the study. In Tampere University Hospital fast protein liquid chromatography (FPLC) was used until June 9, 1999 when the laboratory moved to an immunoassay-based method. In Turku University Hospital the FPLC method was used to until 5 August 1996. Thereafter a high-performance liquid chromatography (HPLC) method was applied and eventually changed to an immunoassay-based method on 1 September 2013. Assays and devices have been described in detail previously (8).

### Oral glucose tolerance tests and glucose assays

Oral glucose tests (1.75 g/kg body weight, up to a maximum of 75 g) were performed according to a standard protocol of overnight fasting, with samples taken at fasting and at 2 h. In Oulu University Hospital capillary samples were used, whereas in Tampere and Turku tests were based on venous samples. In Oulu University Hospital the glucose dehydrogenase-based method was used for the analysis of glucose until May 2000, and thereafter a glucose oxidase method was utilized. In Tampere and Turku the hexokinase method was applied during the whole study. Statistical analyses considering methodological differences are presented in the section on statistical analyses.

### Random plasma glucose assays

Venous plasma samples were obtained in all study sites. In Oulu the glucose dehydrogenase method was used until May 2000 followed by an enzymatic glucose hexokinase method. In Tampere the hexokinase-based method was used until September 2006 and thereafter a glucose dehydrogenase method was applied. In Turku the glucose dehydrogenase method was used during the whole follow-up.

### Definition of dysglycemia

Definitions of dysglycemia have been presented earlier (8, 9). We previously reported predictive HbA1c values based on a 10% rise in HbA1c levels during a 3–12-month interval and HbA1c ≥5.9% (41 mmol/mol) in two subsequent samples. A 10% increase from baseline has been suggested also in other studies (21). In OGTT, cut-offs recommended by WHO for impaired values depending on the method used have been applied and categorized as normal, impaired fasting glucose (IFG), impaired glucose tolerance (IGT), or diabetic. For random plasma glucose a value ≥7.8mmol/l was used, the cut-off being the same as in IGT.

### Statistical analyses

Regarding prevalence of dysglycemia, a total of six different previously defined markers were studied: in the HbA1c material a 10% rise in HbA1c during 3–12 months and two subsequent HbA1c values ≥5.9% (41 mmol/mol); on the OGTT material IFG, IGT, and both IFG and IGT; and in the random plasma glucose material random plasma glucose ≥7.8 mmol/l.

A total of five groups were defined based on the diagnosis of type 1 diabetes and the risk of progression according to autoantibody combinations. Previously the risk of progression has been described in detail, with high risk in children with ≥2 biochemical autoantibodies, significantly lower risk in children with a single autoantibody only, and very low risk if only ICA are detected or transient autoantibodies are observed (6, 19, 22).

The differences in the proportion of dysglycemia in various risk groups were tested with the standardized normal deviate (SND) test. Age at seroconversion and at onset were presented as median (IQR) due to skewed distributions. Glucose parameters were mainly normally distributed in the study population, and means with 95% confidence intervals (CIs) were used. The linear mixed model (LMM) with random intercept and first-order autoregressive (in OGTT series) or unstructured (HbA1c and random plasma glucose) covariance structure for repeated measurements was used to analyze glucose parameters over time between the progressors and non-progressors. The random intercepts and repeated measurements were nested within subjects and subjects within hospital. The group-by-time interaction was included in the model to test differences between group means at each time point. Sex, age at sampling, age at seroconversion and HLA risk were included in the LMM model as fixed variables. All analyses were performed using IBM SPSS Statistic 22.0.0 for Windows and Stata/IC 13.1 for Windows. Figures were drawn using OriginPro 9.1.0 and Stata/IC 13.1.

### Data and resource availability

Anonymized data is available from the corresponding author upon reasonable request. Sharing the data will require additional ethical approval.

## Results

Between November 1994 and August 2014 a total of 18,772 infants with increased genetic risk were enrolled for regular follow-up in the DIPP study. During the follow-up 1162 (6.2% out of all included children) developed at least one islet autoantibody (including ICA). A total of 335 (1.8%) children developed type 1 diabetes. The number of children in each of the five risk groups is shown in **Figure 1**.

Altogether 9912 HbA1c samples, 1935 OGTTs, and 8588 random plasma glucose samples were taken in the five different risk groups (**Figure 1**; **Table 1**). Because of the study design, OGTT tests were not performed in children with ICA only or transient autoantibodies. Baseline characteristics including age, HLA risk, age at seroconversion and duration of follow-up are shown in **Table 1**. Children who later developed clinical type 1 diabetes converted to positivity for islet autoantibodies at a younger age than the other groups (p < 0.001). The frequency of the high-risk HLA genotype was higher among the progressors (**Table 1**).

**Table 1:** Baseline characteristics and frequencies of studied types of dysglycemia in five groups of children with varying risk for type 1 diabetes.

	Progressors^a^	≥2 biochemical autoantibodies^b^	Single biochemical autoantibody^c^	ICA only^d^	Transient^e^
	N = 335	N = 207	N = 189	N = 369	N = 62
Sex, n (%)					
Boys	195 (58)	127 (61)	113 (60)	191 (52)	31 (50)
HLA risk^f^, n (%)					
Neutral or decreased	5 (1)	3 (1)	17 (9)	51 (14)	9 (15)
Low	50 (15)	34 (16)	46 (24)	99 (27)	15 (24)
Moderate	171 (51)	122 (59)	94 (50)	176 (48)	32 (52)
High	109 (33)	48 (23)	31 (16)	43 (12)	6 (10)
Age (years) at seroconversion, median (IQR)	2.0 (1.1–4.0)	3.0 (1.5–5.0)	5.0 (2.5–7.8)	5.1 (2.3–9.0)	2.0 (0.9–6.0)
Age (years) at seroconversion to positivity for multiple (≥2) biochemical autoantibodies, median (IQR)	2.3 (1.5–4.1)	3.8 (2.0–6.9)	NA	NA	NA
Age (years) at T1D^g^ diagnosis or age at the last measurement^h^, median (IQR)	6.3 (3.7–9.3)	10.3 (5.3–14.4)	10.1 (7.6–14.0)	12.0 (9.0–14.9)	8.1 (4.4–12.0)
10% increase in HbA1c values within 3–12 months					
Yes, n (%)	151 (69)	79 (42)	49 (34)	109 (33)	17 (28)
Two consecutive HbA1c values ≥5.9% (41 mmol/mol)					
Yes, n (%)	108 (49)	18 (10)	14 (10)	35 (11)	5 (8)
IFG					
Yes, n (%)	8 (6)	7 (5)	4 (5)	NA	NA
IGT					
Yes, n (%)	74 (35)	22 (14)	2 (3)	NA	NA
Random plasma glucose ≥7.8mmol/l					
Yes, n (%)	76 (26)	9 (5)	9 (5)	14 (4)	2 (3)
Number of HbA1c samples, n	2506	2774	1416	2893	323
Number of OGTTs, n	1025	710	200	NA	NA
Number of random plasma glucose samples, n	2027	1840	1327	3073	321

a The progressor group is defined as children who were diagnosed with type 1 diabetes during the follow-up, regardless of the autoantibody status.

b The ≥2 biochemical autoantibodies group: children who seroconverted persistently positive for ≥2 biochemical autoantibodies but did not develop clinical disease during the follow-up.

c The single biochemical autoantibody group are children who have tested persistently positive for a single biochemical autoantibody only, possibly accompanied by ICA, and who did not develop clinical disease during the follow-up.

d ICA-only group: children who seroconverted to positivity for persistent ICA only and did not develop clinical disease during the follow-up.

e Transient group: children who seroconverted to positivity for any autoantibody but the subsequent test was negative.

f One child in the single biochemical autoantibody group carried a rare HLA genotype not possible to define.

g T1D denotes type 1 diabetes.

h The follow-up ended by the end of August 2014. NA, not available.

### HbA1c

**Figure 2:**
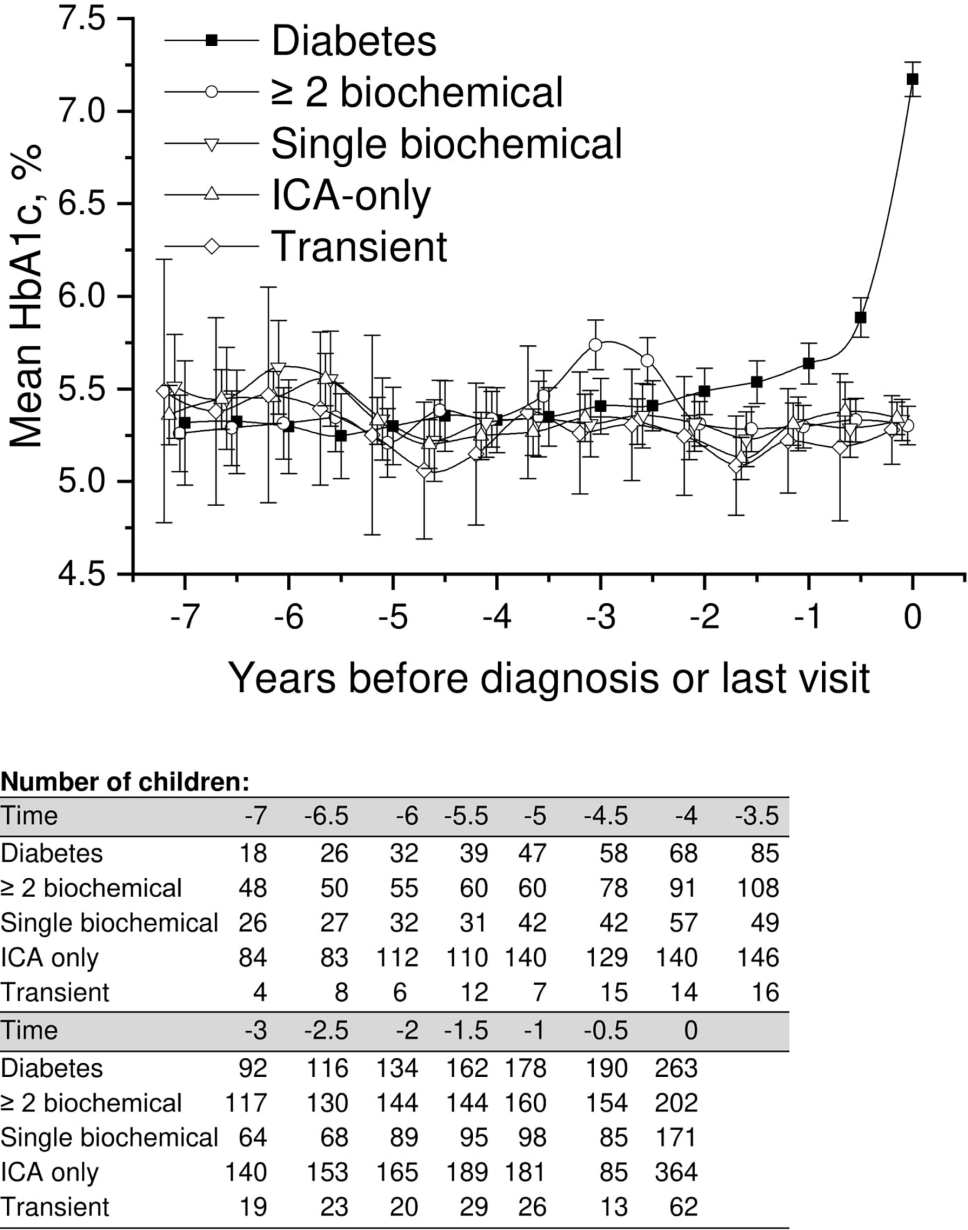
Adjusted mean HbA1c, during the follow-up in children divided into five risk groups. The last points are diagnosis of type 1 diabetes (progressors, black squares) or the last follow-up visit by 31 August 2014. Whiskers show 95% confidence intervals of the adjusted mean in the linear mixed model. In the early values during 4.5–6 years before diagnosis, the progressors showed significantly lower HbA1c values compared to single biochemical autoantibody and low-risk groups. Toward diagnosis, HbA1c values started to increase in the progressors.

We performed a comparison of the HbA1c values in all five groups covering the whole follow-up time, focusing on early differences between the groups. In the LMM analysis we observed a small but significant difference in mean adjusted HbA1c values between progressors and children with a single persistent biochemical autoantibody, ICA only, and those with transient autoantibodies starting 6 years and disappearing 4.5 years before the presentation of type 1 diabetes or before the last visit (**Figure 2**; **Supplementary Table 1**) with lower values observed in the progressors. For example during the early follow up 5.5 to 5.0 years before diagnosis, the mean adjusted HbA1c in the progressors was 5.3% (95%CI 5.2–5.4) [34 mmol/mol (95%CI 33–36)] compared to the single autoantibody group 5.6% (95%CI 5.5–5.7) [38 mmol/mol (95%CI 37–39); p < 0.001], those with ICA only 5.6% (95%CI 5.6–5.7) [38 mmol/mol (95%CI 38-39); p < 0.001], and those with transient autoantibodies 5.5% (95%CI 5.3–5.6) [37mmol/mol (95%CI 34–38); p = 0.026].

A similar but a slightly weaker difference was observed between children with multiple biochemical autoantibodies and the low-risk groups. Analogous with progressors, HbA1c values were slightly but significantly lower than in other groups (**Figure 2**; **Supplementary Table 1**). Exact crude values from 7 years before diagnosis or last visit are provided in the Supplementary material with 0.5-year intervals.

### Oral glucose tolerance test

**Figure 3:**
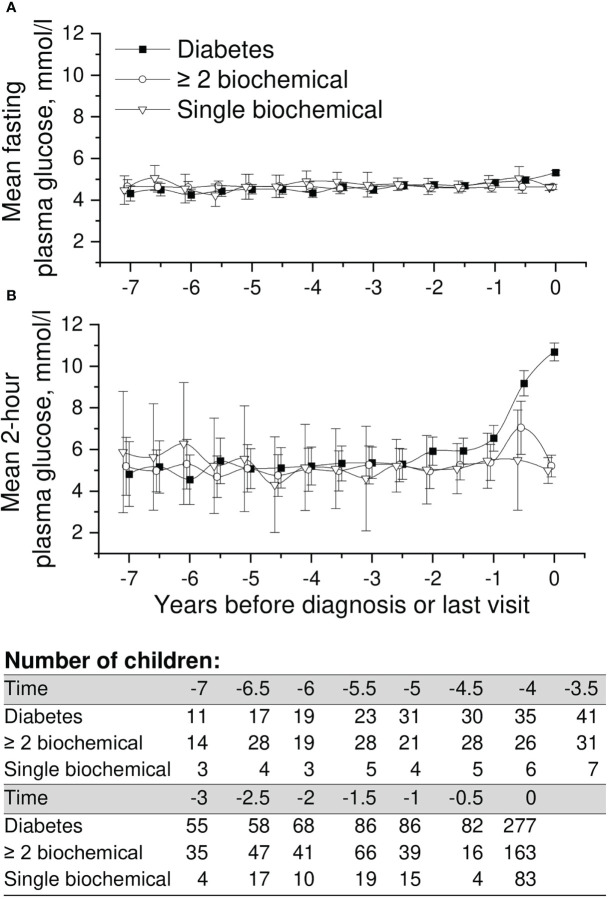
Adjusted mean plasma glucose levels in OGTT [**(A)** =fasting and **(B)** =2-h plasma glucose], during the follow-up in children in three risk groups (OGTTs were not performed in the ICA only and transient autoantibody positivity groups). The last points are diagnosis of type 1 diabetes (progressors, black squares) or the last follow-up visit by August 31, 2014. Whiskers show 95% confidence intervals of the adjusted mean in the linear mixed model. No significant differences in the early plasma glucose values were observed between the groups. OGTT values started to rise toward diagnosis in the progressors.

Mean adjusted fasting plasma glucose and 2-h values in OGTT in the three groups available are presented in **Figure 3**. We observed no differences in the early glucose values between the groups. As assumed, 2-h values started to rise toward diagnosis in progressors, whereas fasting plasma glucose values remained stable until the day of diagnosis. Exact crude values are provided in the Supplementary material (**Supplementary Table 1**).

### Random plasma glucose

**Figure 4:**
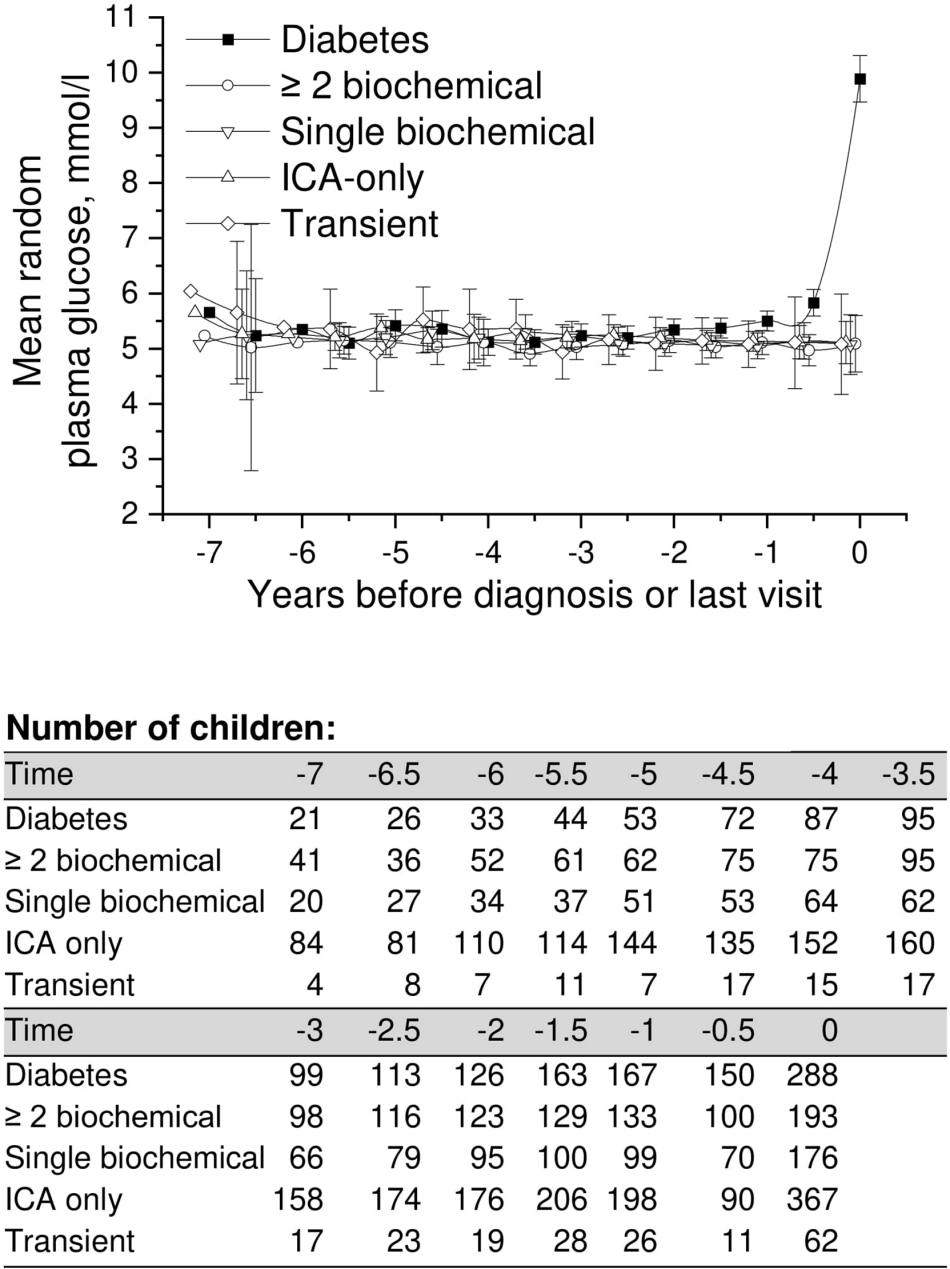
Adjusted mean random plasma glucose during the follow-up in children in five risk groups. The last points are diagnosis of type 1 diabetes (progressors, black squares) or the last follow-up visit by August 31, 2014. Whiskers show 95% confidence intervals of the adjusted mean in the linear mixed model. No significant differences in the early random plasma glucose values were observed between the groups. Random plasma glucose values started to increase toward diagnosis in the progressors. .

Mean adjusted random plasma glucose showed no differences in the early follow-up phases between the five groups compared (**Figure 4**). Similar to other parameters analyzed (HbA1c, OGTT), values started to rise toward the diagnosis in progressors. Exact crude values are provided in the Supplementary material (**Supplementary Table 1**).

### Appearance of dysglycemic markers in the five risk groups

Earlier identified markers of dysglycemia were compared between the five groups: In the HbA1c series, a 10% rise in HbA1c during 3–12 months and two subsequent HbA1c values ≥5.9% (41 mmol/mol); in the OGTT series IFG and IGT; and in the random plasma glucose series plasma glucose ≥7.8 mmol/l are presented in **Table 1**. As reported earlier (8, 9), these markers are common prior to the diagnosis of type 1 diabetes. The frequency of any sign of dysglycemia was 66% in progressors, 46% in those with ≥2 islet autoantibodies, 31% in those with a single autoantibody, 27% in those with ICA only, and 31% in those with transient autoantibodies. If excluding the 10% increase during 3–12 months in HbA1c with the lowest specificity observed (**Table 1**), the proportions were 55%, 21%, 11%, 13% and 9%, respectively.

Sensitivities and specificities of the markers of dysglycemia studied are as follows: a 10% rise in HbA1c values during 3–12 months 69% and 58%, two subsequent HbA1c values ≥5.9% (41 mmol/mol) 49% and 91%, IFG 6% and 95%, IGT 35% and 86%, and random plasma glucose ≥7.8mmol/l 26% and 95% when including children with the highest risk with ≥2 biochemical autoantibodies (n = 499) (**Table 1**; **Figure 1**).

### Glucose values in low-risk groups

Only three children (**Figure 1**) developed type 1 diabetes during the follow-up in the group with ICA only (0.3%) and in the group with transient autoantibodies (3%). The risk for diabetes was lower in the ICA-only group than in the general population (approximately 0.8% by the age of 15 years), and the risk of the transient group was comparable to that associated with HLA risk only. HbA1c values during the whole follow-up varied between 5.2% (33mmol/mol) and 5.6% (38mmol/mol). In random plasma glucose values variation was smaller than in HbA1c. Plasma glucose values in the ICA-only group were constantly between 5.1 mmol/l and 5.3 mmol/l during the 5.5-year follow-up period before the last visit. In the transient group values varied between 4.9 and 5.5 mmol/l (**Figures 2**, **4**). For exact values during follow-up, see **Supplementary Table 1**.

## Discussion

The prediction of type 1 diabetes has improved significantly in the past years along with increasing knowledge of islet autoantibodies and glucose metabolism. Recently the possibility of an early defect in pancreatic development during organogenesis has been implicated. In the current study we characterized glucose metabolism in children at genetic risk who were observed within the Finnish DIPP study from 1994 to 2014 with a total sample size of 1162 children. Risk groups were formed according to autoantibody combinations. For the first time we describe glucose metabolism in a large group of low-risk children, seroconverting to positivity for ICA only (0.3% developed type 1 diabetes) or with transient autoantibodies (3% developed type 1 diabetes). We observed lower early HbA1c values in future progressors compared to the low-risk groups 6.0 to 4.5 years before their diagnosis or last visit. This finding, however, was not supported by other glucose parameters, since no differences were observed in the early glucose values in OGTT or random plasma glucose and there is a possibility of a chance finding. We also confirmed a high incidence of dysglycemia in children progressing to type 1 diabetes during follow-up. Especially, two consecutive HbA1c values ≥5.9% (41 mmol/mol) seem practical in the identification of future patients. Those definitions of dysglycemia have been used previously by us (8, 9, 23) and others (7, 24).

A clear strength of the study is the unique design of the DIPP study, follow-up since birth, and screening for islet autoantibodies. We were able to define autoantibody combinations and divide participants into five groups according to diabetes risk (19). Previously an IVGTT study comparing progressors and children positive for ICA only has been published from the DIPP study, suggesting an early defect in β-cell function (11). The large series of children studied here and the high number of HbA1c, OGTT, and random glucose analyses preformed over a long follow-up period allowed us to provide conclusions on glucose metabolism in different risk groups and generate information on normal levels in low-risk children, comparable to the general population in relation to the risk of type 1 diabetes. Our study population came from three clinical centers where slightly different methods to analyze HbA1c, plasma glucose during the OGTT, and random plasma glucose were used. This could produce slightly different values, thus affecting our results. However, this potential confounding factor affects all groups similarly, and different hospitals, long time period, and varying age of the study participants have been taken into account in the statistical analyses. Furthermore, although some differences existed, the used laboratory methods are common and generalizable to other populations with commonly used cut-off values. One source of confounding is the small number of participants with long follow-up time before diagnosis or last visit. In these children the disease process can be different from those with fast progression from seroconversion to type 1 diabetes, reducing the generalizability of our findings.

In the current study, HbA1c values were lower in future progressors than in the low-risk groups. This is in disagreement with the previous finding based on IVGTT, reporting lower FPIR values in progressors. A parallel finding was neither seen in OGTT or random plasma glucose values. The sensitivity of OGTT is however affected by annual sampling, where HbA1c and random plasma glucose were obtained every 3-months. Also an analogous finding of lower HbA1c levels was observed in children with multiple biochemical autoantibodies not progressing to type 1 diabetes during follow-up. This suggests an actual difference in early glucose metabolism between high- and low-risk individuals. The reduced HbA1c values several years before disease manifestation in the progressors indicate that such children may have lower average glucose concentrations in their peripheral circulation than the non-progressors early in life. This would not necessarily be picked up by an OGTT or by random glucose measurements every third month. The observation implies that those children who later present with type 1 diabetes may have variable glucose metabolism in their early years. Although reasons behind this remain speculative, previous studies have shown alterations also in glucagon-producing alpha cells in preclinical type 1 diabetes (25). It remains possible that alpha cell dysfunction is presently lost prior to decreasing insulin secretion leading to hypoglycemia in future progressors.

As reported before, we show a high rate of dysglycemia in children progressing to type 1 diabetes during follow-up. Very high specificity for developing type 1 diabetes is observed for IFG, IGT, and random plasma glucose ≥7.8mmol/l, although with low sensitivity, as reported earlier (9). A 10% increase in HbA1c values during a time period of 3–12 months shows higher sensitivity with compromised specificity. As suggested before (8), among the parameters studied, two consecutive HbA1c values ≥5.9% (41 mmol/mol) seem most adequate in the prediction of future progressors with 49% sensitivity and 91% specificity.

A series of measures have been tested for the prevention of type 1 diabetes before the appearance of autoantibodies (primary prevention), after the detection of islet autoantibodies (secondary prevention) and after the presentation of clinical disease (tertiary prevention) with no persistent success (12). The reasons for these undesirable results have been discussed and some evidence for different subtypes of the pathogenic process has been proposed, e.g., more aggressive insulitis in younger individuals (26). Different proportions of lymphocytes (27) and, in some patients, more general inflammation in the pancreas (28) have been observed, possibly being a consequence of the effect of genetic factors or predisposition to infectious agents (29). It is possible that early development and maturation of the pancreas in individuals that are destined to develop type 1 diabetes predisposes these patients to a high probability of disease development that is hard to avoid.

The normal volume of the pancreas is around 90 cm^3^ in adults, with islets accounting for 1%–2% of this. In cadaveric pancreases the actual β-cell mass varies up to fivefold in healthy individuals independent of age and BMI (30). Pancreatic mass seems to be frequently smaller in patients with recent-onset diabetes and, worth noting, also in autoantibody-positive subjects without overt disease, compared to healthy controls (15, 16). This implies deficiencies already in the early development.

According to our results, glucose values in children with low risk of diabetes remain relatively stable. HbA1c is possibly lower in early life in future progressors compared to low-risk groups of children. Dysglycemia is common before the presentation of clinical type 1 diabetes, and especially, increasing HbA1c values can be used in predicting disease or potentially as an inclusion criterion in future trials aimed at postponing or preventing the development of type 1 diabetes.

## Data availability statement

Anonymised data are available from the corresponding author upon reasonable request. Sharing the data will require additional ethical approval.

## Ethics statement

The studies involving human participants were reviewed and approved by Ethics committee of Northern Ostrobothnia. Written informed consent to participate in this study was provided by the participants’ legal guardian/next of kin.

## Author contributions

Contribution statement OH had full access to all of the data in the study and takes responsibility for the integrity of the data and the accuracy of the data analysis. Study concept and design: OH, JI, OS, MK, RV. Acquisition of data: OH, SA, JI, MK, RV. Analysis and interpretation of data: OH, TP, MK, RV. Drafting of the manuscript: OH, TP, MK, RV. Critical revision of the manuscript for important intellectual content: OH, TP, SA, JI, OS, MK, RV. Statistical analysis: TP. Administrative, technical, or material support: OH, TP, JI, MK, RV. Study supervision: RV. All authors have read and approved the final version.

## Funding

This work was supported by the following grants. International: JDRF International (grants 4-1998-274, 4-1999-731, 4-2001-435); European Union (grant BMH4-CT98-3314); Novo Nordisk Foundation. Finland: Academy of Finland (Centre of Excellence in Molecular Systems Immunology and Physiology Research 2012-2017, Decision No. 250114); TEKES National Technology Agency of Finland; Special Research Funds for University Hospitals in Finland; Finnish Office for Health Technology Assessment; Diabetes Research Foundation, Finland; Sigrid Juselius Foundation; Emil Aaltonen Foundation; Jalmari and Rauha Ahokas Foundation; Signe and Ane Gyllenberg Foundation; the Research Foundation of Orion Corporation; Foundation for Pediatric Research; Alma and KA Snellman Foundation; Päivikki and Sakari Sohlberg Foundation, the Finnish Medical Foundation and Finnish Cultural Foundation, North Ostrobothnia Regional Fund. The funders were not involved in the study design, collection, analysis, interpretation of data, the writing of this article, or the decision to submit it for publication. All authors declare no other competing interests.

## Acknowledgments

We thank the dedicated personnel of the DIPP study in Oulu, Tampere and Turku; and the study children and their families for their essential contribution.

## Conflict of interest

The authors declare that the research was conducted in the absence of any commercial or financial relationships that could be construed as a potential conflict of interest.

## Publisher’s note

All claims expressed in this article are solely those of the authors and do not necessarily represent those of their affiliated organizations, or those of the publisher, the editors and the reviewers. Any product that may be evaluated in this article, or claim that may be made by its manufacturer, is not guaranteed or endorsed by the publisher.
