# Supplementary material for: Early glucose metabolism in children at risk for type 1 diabetes based on islet autoantibodies compared to low-risk control groups
Source: Front Endocrinol (Lausanne). 2022 Sep 12;13:972714. doi: 10.3389/fendo.2022.972714 (PMC9511031; doi:10.3389/fendo.2022.972714)
Supplement: Supplementary file 1 [file DataSheet_1.docx]

**Supplementary material**

Supplement to:

Helminen O, Pokka T, Aspholm S et al. Early Glucose Metabolism in Children at Risk for Type 1 Diabetes Based on Islet Autoantibodies Compared to Low-Risk Control Groups

Contents:

This Supplementary material presents the mean adjusted values and 95%CIs of HbA1c, OGTT and random plasma glucose used in this study during follow-up until diagnosis of type 1 diabetes or last visit.

Supplementary Table 1
